# Mixed Dimensional ZnO/WSe_2_ Piezo-gated Transistor with Active Millinewton Force Sensing

**DOI:** 10.1021/acsami.2c15730

**Published:** 2022-10-19

**Authors:** Yulin Geng, Jing Xu, Muhammad Ammar Bin Che Mahzan, Peter Lomax, Muhammad Mubasher Saleem, Enrico Mastropaolo, Rebecca Cheung

**Affiliations:** † Institute for Integrated Micro and Nano Systems, School of Engineering, University of Edinburgh, Scottish Microelectronics Centre, Edinburgh EH9 3FF, United Kingdom; ‡ Department of Mechatronics Engineering, National University of Sciences and Technology (NUST), Islamabad 44000, Pakistan

**Keywords:** piezo-gated field-effect transistors, force sensors, low dimensional materials, ZnO nanorods, 2D materials, piezoelectric effect

## Abstract

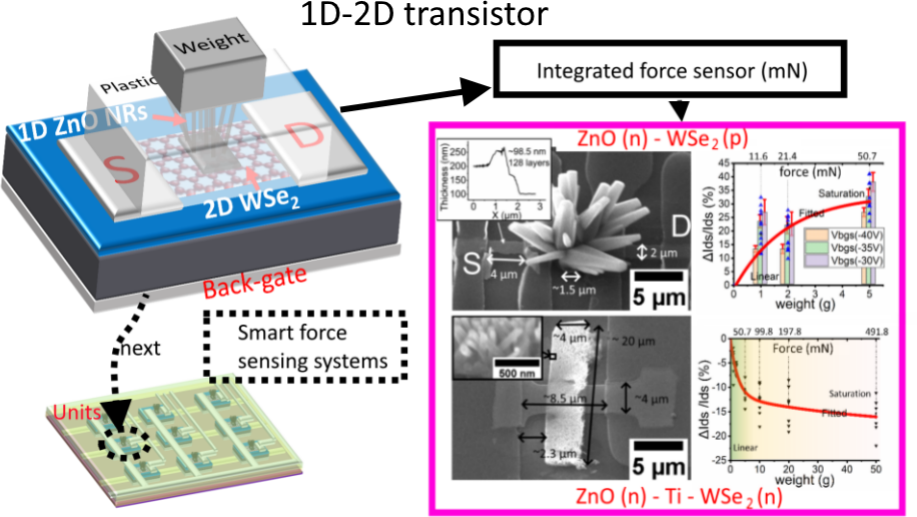

This work demonstrates a mixed-dimensional piezoelectric-gated transistor in the microscale that could be used as a millinewton force sensor. The force-sensing transistor consists of 1D piezoelectric zinc oxide (ZnO) nanorods (NRs) as the gate control and multilayer tungsten diselenide (WSe_2_) as the transistor channel. The applied mechanical force on piezoelectric NRs can induce a drain–source current change (Δ*I*_ds_) on the WSe_2_ channel. The different doping types of the WSe_2_ channel have been found to lead to different directions of Δ*I*_ds_. The pressure from the calibration weight of 5 g has been observed to result in an ∼30% *I*_ds_ change for ZnO NRs on the p-type doped WSe_2_ device and an ∼−10% *I*_ds_ change for the device with an n-type doped WSe_2_. The outcome of this work would be useful for applications in future human–machine interfaces and smart biomedical tools.

## 1 Introduction

With the rapid development of the Internet of Things (IoT) and complex human–machine interfaces, much attention has been paid to the miniaturization of the force-sensing units in the past few decades.^1−4^ Apart from providing the possibility for a higher integration level and a higher spatial resolution,^1,5,6^ because of a smaller effective loading area, the smaller force-sensing unit can be used to detect the smaller tactile force in the millinewton or micronewton range. The small force detection could be helpful in providing feedback during the manipulation of micro-objects.^7^ One example of the small force sensor application is the assistant tool for precise surgery, such as ophthalmological surgery, when the tactile force is critical for the safety and outcomes of surgery operations.^8,9^

In the past few decades, much effort has been made to minimize the capacitive, piezoelectric, and piezoresistive components as the loading units of mechanical force sensors.^7,10,11^ However, currently, most of the force-sensing units are passive. The passive signals depend only on the functional structure or materials and cannot be controlled without an external system. For an active sensing system, one could control the signal by applying an additional signal to provide smarter and more adaptive sensing functions.^5,12,13^ In the literature, to achieve the adaptive force sensing, the integration of the force-sensing materials or structures onto a field-effect transistor (FET) has been treated as one of the possible solutions.^2,3,5,14^ With a higher integration level, the transistor-type force sensor could be more compatible with the current CMOS (Complementary Metal Oxide Semiconductor) system, and then the advanced programming algorithm such as machine learning can be implemented,^15^ allowing more complex functions and more adaptive systems to be achieved.^15−17^

**Figure 1:**
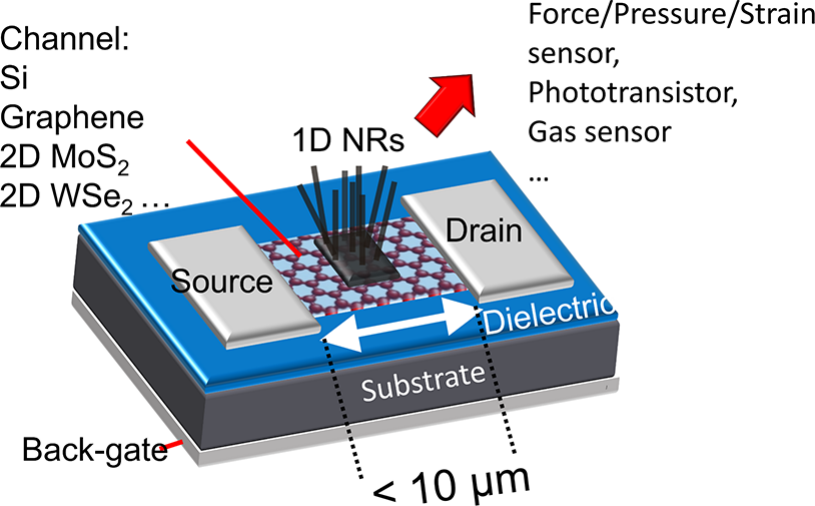
Schematic showing mixed-dimensional transistor as an active sensing unit. The channel materials can be doped Si (MOSFET, metal-oxide-semiconductor FET),^21−23^ graphene,^24−26^ and 2D MoS_2_.^14^ Normally, the piezoelectric ZnO NRs could be employed as a gate to achieve force/pressure/strain sensing. In addition to force sensing, mixed-dimensional transistors have been reported to have capabilities in photodetection^23,25,26^ and gas sensing.^32^

The development of one-dimensional (1D) piezoelectric nanomaterials such as zinc oxide (ZnO) and gallium nitride (GaN) nanorods (NRs) provides the possibility to achieve the piezoelectric based force sensor in the microscale.^18−20^ These piezoelectric nanomaterials can be integrated with a FET as the gate control (see Figure 1): when pressure is loaded on the piezoelectric material, there will be a drain–source current change (Δ*I*_ds_) in the FET channel. The channel of FET can be made by different materials, such as doped silicon (Si),^21−23^ graphene,^24−26^ and two-dimensional (2D) molybdenum disulfide (MoS_2_).^14^ Among these channel materials, 2D materials have drawn much interest due to a higher carrier mobility in the “short channel” scale, which could offer more potential for the field effect to amplify the electrical signal when the device geometry is scaled down further.^27,28^ With the 1D–2D integrated structure, the small pressure from the normal direction can induce a large strain on the vertically grown piezoelectric NRs due to the high aspect ratio in the *z*-axis, leading to a large polarization. The polarization could be amplified by the 2D semiconductor channel with a back-gate voltage. Overall, the mixed-dimensional transistor could be a potential way to sense the small pressure and amplify the piezoelectric signal by itself. The output signal (drain–source current *I*_ds_) is more compatible with the CMOS system to achieve more complex and adaptive functions. In addition, with a self-powered piezoelectric gate-control block,^29−31^ the sensing system based on the piezo-gated transistor could have an improved power efficiency in the overall sensing system. However, the development on these mixed-dimensional transistors is still in its infancy; the study of the mechanism of these novel structures is still limited, and the integration and the fabrication process of these novel mixed-dimensional transistors still require much effort.

This paper demonstrates a piezo-gated force-sensing transistor that consists of a vertical one-dimensional (1D) ZnO NR array and a multilayer tungsten diselenide (WSe_2_) channel (1D–2D transistor, see Figure 1). The ZnO NR array has been selected as the piezo-gate of the transistor, mainly because of the relatively high sensitivity to pressure (piezoelectric coefficient *d*_33_ up to 9.5 pC/N^33^) and the relatively simple synthesis methods.^34,35^ The reasons to select the WSe_2_ as the channel materials are that (1) the doping level of WSe_2_ can be modified,^36−38^ which gives more possibility for tuning the *I*_ds_ in the channel , and (2) the breaking strain of WSe_2_ is relatively high, which could be helpful in future flexible substrates.^39^ In this work, two example microscale devices have been presented that show the different directions of *I*_ds_ change and different sensitivities (up to 30% *I*_ds_ change) under pressure loading by calibration weights (equivalent to the millinewton range force). The piezo-gated mechanism and the factors that influence the force-sensing performance have been discussed. The integration of ZnO NRs on both p-type and n-type doped WSe_2_ transistors and the successful detection of two different direction changes of *I*_ds_ under pressure could demonstrate the potential of the 1D–2D transistor for smart force-sensing applications in the future, such as human–machine interfaces and biomedical applications.

## 2 Results and Discussion

### 2.1 Piezo-Gated Transistor with a p-Type Doped WSe_2_ Channel (Dev. 1)

#### 2.1.1 Fabricated Device (Dev. 1) and the Testing Setup

**Figure 2:**
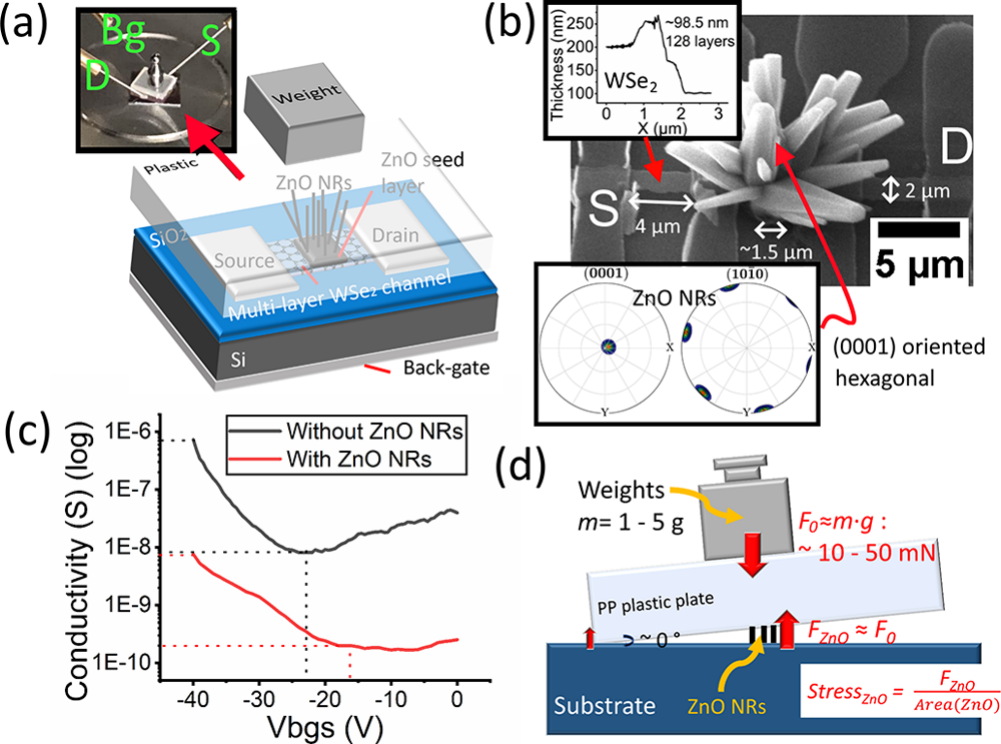
(a) Device schematic of the ZnO-WSe_2_ piezo-gated transistor (inset with the device testing setup). (b) SEM images of exampled ZnO-WSe_2_ piezo-gated transistor (inset with AFM profile showing the thickness of the WSe_2_ flake, together with the EBSD pole figure showing the crystal (0001) orientation of ZnO NRs grown on the ZnO seed layer, adapted from ref (40) and used under Creative Commons CC-BY 3.0 license). (c) Sheet conductivity of the WSe_2_ channel before and after ZnO NRs growth. (d) Schematic showing the input force and the stress applied on the ZnO NR array.

An example device (Dev. 1) with ZnO NRs flower grown on the p-typed doped WSe_2_ transistor has been fabricated (for details see Materials and Methods and Supporting Information Section S1) and is presented in Figure 2. For the example device, mechanical exfoliated multilayer WSe_2_ (∼128 layers) has been used as the transistor channel, and the patterning of the channel (2 μm × 4 μm) and the metal contact (100 nm thick titanium (Ti)) have been achieved by photolithography. The WSe_2_ channel layer has been overetched by XeF_2_ vapor, which has been reported to induce the defect on the etching edge and could possibly lead to a p-type doping behavior.^38^ The integration of the vertical ZnO NR array (diameter ∼ 1 μm, length ∼ 6 μm, flower cluster shape array on WSe_2_ channel, Figure 2b) has been achieved by patterned hydrothermal growth on an e-beam evaporated ZnO seed layer.^40^ On the basis of our previous characterization by electron backscatter diffraction (EBSD), the vertical ZnO nanorod grown hydrothermally on e-beam evaporated ZnO seed layer possesses a single crystalline with (0001) orientation along the *z*-axis (see the inset of Figure 2b).^40^

The measurement of the transistor has been performed by the semiconductor analyzer Keithley 4200, and the testing setup can be seen in the inset of Figure 2a. To study the influence of the integration process of ZnO NRs on the electric properties of the WSe_2_ channel, the sheet conductivity of the transistor channel has been measured before and after the integration of ZnO NRs under different back-gate voltages (*V*_bgs_), and the results are presented in Figure 2c. It can be observed that the deposition of the ZnO seed layer and ZnO NRs has decreased the conductivity of the WSe_2_ transistor by a factor of ∼10^2^ (see Figure 2c), and the back-gate threshold voltage of the overall transistor has been observed to shift from −23 V to −16 V (see Figure 2c). The decrease of the overall conductivity and the shifting of the threshold voltage could be explained by the additional surface scattering on the top side and the possible oxidation of WSe_2_ (more holes, equivalent to p-type doping)^41^ due to the deposition of ZnO seed layer/NRs.

The input force on the devices has been performed by the calibration weights, and a polypropylene (PP) plastic plate (1 cm^2^, 2 mm thick, ∼0.18 g) has been used for encapsulation and mechanical force coupling (Figure 2a, inset). Calibration weights (1 g, 2 g, 5 g) have been used to apply a mechanical load on the 1D–2D transistor, which is equal to the force of ∼11.6 mN, ∼21.4 mN, and 50.7 mN, respectively (*F* = *m* × *g*, taking *g* = 9.8 N/kg as the standard gravity, including the PP plastic plate). As shown in Figure 2d, the ZnO NR area is close to the center of gravity of the overall load, and the length of ZnO NRs (∼6 μm) is significantly shorter than the width of the PP plastic plate. Therefore, the force applied on ZnO NRs is assumed to be the same as the overall force from the gravity of the weights and the PP plastic plate. The area of ZnO NRs for Dev .1 is around 15 μm^2^ (∼10 μm × 1.5 μm), the applied forces by calibration weights (1 g, 2 g, 5 g) are equivalent to the stresses of 0.77, 1.43, and 3.39 GPa on ZnO NRs. However, it is worth noting that the exact strain on ZnO NRs could be significantly lower due to the unstable mechanical coupling and strain sharing by the substrate.

#### 2.1.2 Force-Sensing Characterization of Dev. 1

**Figure 3:**
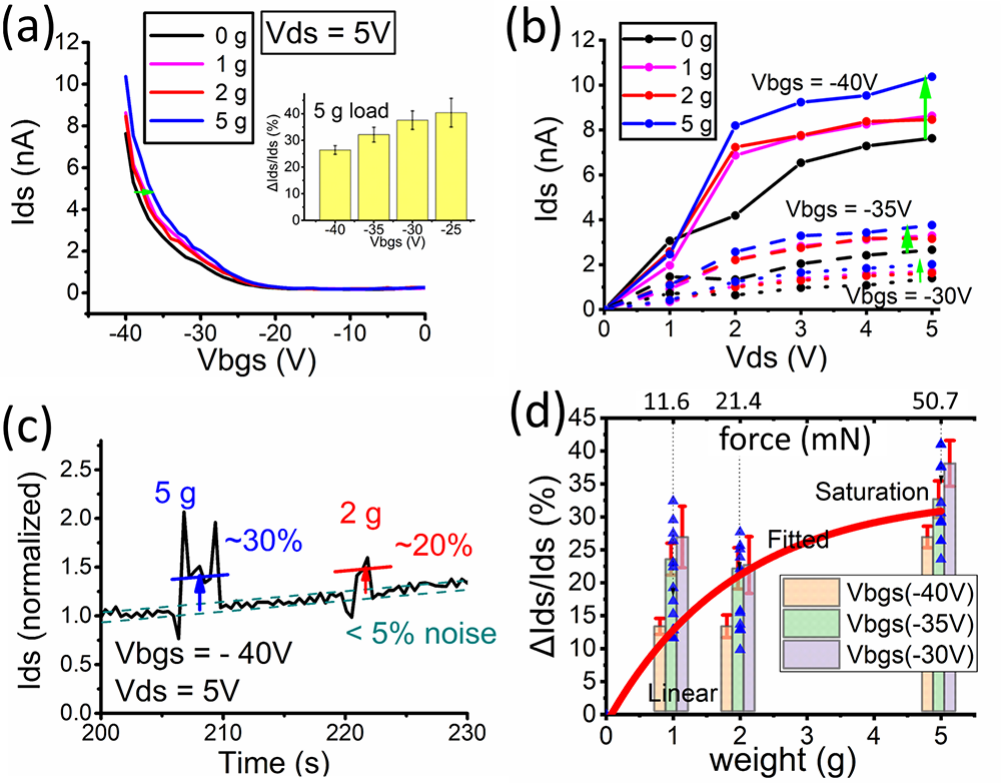
(a) Back-gate transfer characteristics of the ZnO-WSe_2_ piezo-gated transistor as different weights are loaded, inset with the bar diagram that shows the (Δ*I*_ds_/*I*_ds_)% under a 5 g load with *V*_bgs_ ranging from −40 V to −25 V. (b) Output characteristics of the ZnO-WSe_2_ piezo-gated transistor. (c) Real-time sensing measurement of the transistor with 2 and 5 g of weight loading and *I*_ds_ normalized (the unloaded *I*_ds_ has been set as 1). (d) Drain–source current change ((Δ*I*_ds_/*I*_ds_)%)) as a function of applied weight and estimated pressure (with data scatter plots); in addition, the bar diagram shows the (Δ*I*_ds_/*I*_ds_)% under *V*_bgs_ between −40 V to −30 V.

After the integration of ZnO NRs, the drain–source current (*I*_ds_) of the overall transistor can be controlled by the back-gate voltage (*V*_bgs_), the drain–source voltage (*V*_ds_), and the applied mechanical load (weights). The transfer and output characteristics of the overall transistor after integration of ZnO NRs can be seen in Figure 3a,b.

Without a mechanical load (0 g), the overall transistor is similar to a long channel depletion PMOS transistor. The overall transistor is “closed” when the *V*_bgs_ is between ∼−20 and 0 V. When the back-gate voltage is more negative than ∼−20 V, the *I*_ds_ increases up to a few nanoamperes (see transfer characteristics in Figure 3a). The back-gate control (*V*_bgs_) is based on the field effect. The negative voltage induces more holes (majority carriers) in multilayer WSe_2_ to the bottom by an electric field through 300 nm thick SiO_2_. Holes accumulate in the first few bottom layers of WSe_2_ or the interface between the bottom layers of WSe_2_ and the back-gate dielectric (300 nm thick SiO_2_), which is believed to form the p-type channel. For the output characteristic (Figure 3b), the *I*_ds_ has been observed to be saturated when the *V*_ds_ values are larger than 3 V, which is similar to the pinch-off mechanism of the MOSFET. The *I*_ds_ seems relatively unstable compared to the high performance 2D WSe_2_ FETs in the literature.^42^ The reason could be the high contact resistance between Ti and p-type doped WSe_2_ (Schottky contact)^43^ and the surface scattering between SiO_2_ and WSe_2_.^44^

After the weight loading (1–5 g), a positive shift of 2–5 V in *V*_bgs_ has been observed for the overall transfer curve (Figure 3a). As for the output characteristics (Figure 3b), when the *V*_ds_ value is over 2 V, it can be observed that the loading weights can result in an apparent increase in the level of *I*_ds_. Overall, the effects of applied weights to Dev. 1 contribute to an increasing *I*_ds_. The sensitivity of weight sensing has been observed to be different when the different *V*_bgs_ values have been applied (see inset of Figure 3a). When the applied *V*_bgs_ changes from −25 V to −40 V and the load has been fixed at 5 g, the percentage change of *I*_ds_ ((Δ*I*_ds_/*I*_ds_)%) has been observed with a decreasing trend as *V*_bgs_ becomes more negative (see inset of Figure 3a). It is worth noting that the 1 g load seems to have a larger output than the 2 g load, as can be seen in Figure 3a,b, which could be explained by the hysteresis between deformation and recovery of the piezoelectric NRs (loading sequence 2 g, 5 g, and then 1 g; see Supporting Information Section S2).

When the PP plastic plate (0.18 g, ∼1.7 mN) is loaded on the device, a relatively large current change has been observed (from 6 nA to 7.5 nA, *V*_bgs_ = −40 V, *V*_ds_ = 5 V, see Supporting Information Section S2), which may indicate that the minimum detection limit of the transistor could be potentially less than several millinewtons. For the maximum loading limit, it has been observed that ZnO NRs (fraction strength from 2.9 to 8.1 GPa^45^) can stand for a 5 g load without breaking. During the heavier weights (over 10 g) loading, the device has been observed to be broken. However, based on the optical image after the breaking of the device (see Supporting Information Section S2), apart from overloading, the shear force between the plastic plate and ZnO NRs during loading could be another reason for the breaking since the ZnO-WSe_2_ has been observed to be peeled from the substrate.

The real-time force-sensing test has also been performed. The *I*_ds_ has been observed with ∼30% and ∼20% increase under 5 and 2 g of loading, with a relatively high signal-to-noise ratio (Figure 3c). It is worth noting that the *I*_ds_ has been found to be sensitive to light intensity, which is believed to be one of the reasons for the shifting of the real-time *I*_ds_ curve (Figure 3c); more details have been provided in Supporting Information S3. In addition, the relationship between the applied weights and the percentage change of *I*_ds_ ((Δ*I*_ds_/*I*_ds_)%) has been plotted with scatter data points (Figure 3d). The trend for the *I*_ds_ change has been observed to have a larger increasing slope and a quasi-linear behavior at low pressure (0–1 g) and then saturatation as the applied pressure increases (2–5 g). The overall relationship has been found to be similar to the piezoelectric output curve of the ZnO NRs-MOS capacitor device and ZnO NRs-2D MoS_2_ devices.^14,46^ The explanation for the nonlinear relationship between force and the *I*_ds_ change could be the possible dominant role of contacting/triboelectric charge under low pressure and the saturation of the stress–strain behavior of ZnO NRs within such a small area.^14,46^

#### 2.1.3 Force-Sensing Mechanism of Dev. 1

**Figure 4:**
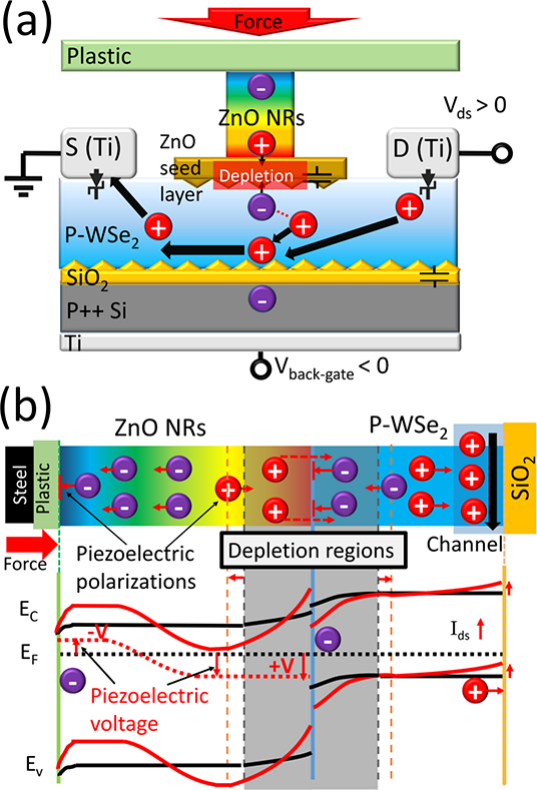
(a) Working mechanism schematic of the piezo-gated transistor. (b) Energy band diagram showing the p–n junction control mechanism (the red curves are the conduction band, Fermi level, and valence band after applying pressure on ZnO).

The mechanism of the overall transistor has been discussed with the help of the structure diagram (Figure 4a) and the energy diagram (Figure 4b). The overall working mechanism of Dev. 1 is as follows:

(1) Without pressure, the channel of holes is formed in the bottom side of p-type doped WSe_2_ by the field-effect from the back-gate voltage (see Figure 4a). It is worth noting that even the top sides of the ZnO NRs are floated, and there could be a formation of the semiconductor junction between n-type doped ZnO NRs (due to the oxygen-related defects^47^) and p-type doped WSe_2_ (overetched by XeF_2_^38^). Once ZnO is in contact with WSe_2_, the majority carriers from both WSe_2_ (holes) and ZnO (electrons) could diffuse to each other as a result of the concentration gradient. A depletion region (see Figure 4b) could be formed when the thermodynamical equilibrium has been reached such that the inner electrostatic force is the same as the diffusion force. The existence of the depletion region could be one of the reasons for the degradation of the channel conductivity and the decreasing of *I*_ds_ after the ZnO NRs have been grown on WSe_2_ (see Figure 2c). However, the study of the depletion region between two different low-dimensional materials is still under investigation.
(2) When pressure is applied on the ZnO NRs, the dipoles of ZnO NRs could be compressed, which is equivalent to a positive piezoelectric polarization (along the *z*-axis) between the bottom and the top side of the ZnO NR.^48^ The top sides of ZnO NRs are connected with PP plastic (dielectric) and band-aligned with the iron (calibration weights, virtual grounded) after contacting. The piezoelectric polarization (positive charge) on the bottom side of ZnO could be equivalent to applying a positive voltage on the n-side of the p–n junction, which prevents the majority carriers from flowing through the junction (see Figure 4b). Then, the positive piezoelectric voltage can drive more electrons in WSe_2_ (minority carrier) accumulated on the top of WSe_2_ and repel more holes of the WSe_2_ (majority) carrier to the bottom of WSe_2_ (the hole conducting channel). Therefore, the overall current *I*_ds_ in the WSe_2_ channel increases. In addition, the increasing *I*_ds_ can also be possibly due to the minority carrier (holes) injection from the bottom of the ZnO NRs to the WSe_2_ channel driven by piezoelectric voltage.
(3) The observation that, at 5 g, the ((Δ*I*_ds_/*I*_ds_)%) decreases as *V*_bgs_ becomes more negative (see inset of Figure 3a) suggests the higher the hole concentration in the channel to begin with at high negative *V*_bgs_, the weaker the modulation of the channel from the effective gate voltage resulting from the 5 g weight.

### 2.2 Piezo-Gated Transistor with an n-Type Doped Channel and a Ti Intermediate Layer (Dev. 2)

**Figure 5:**
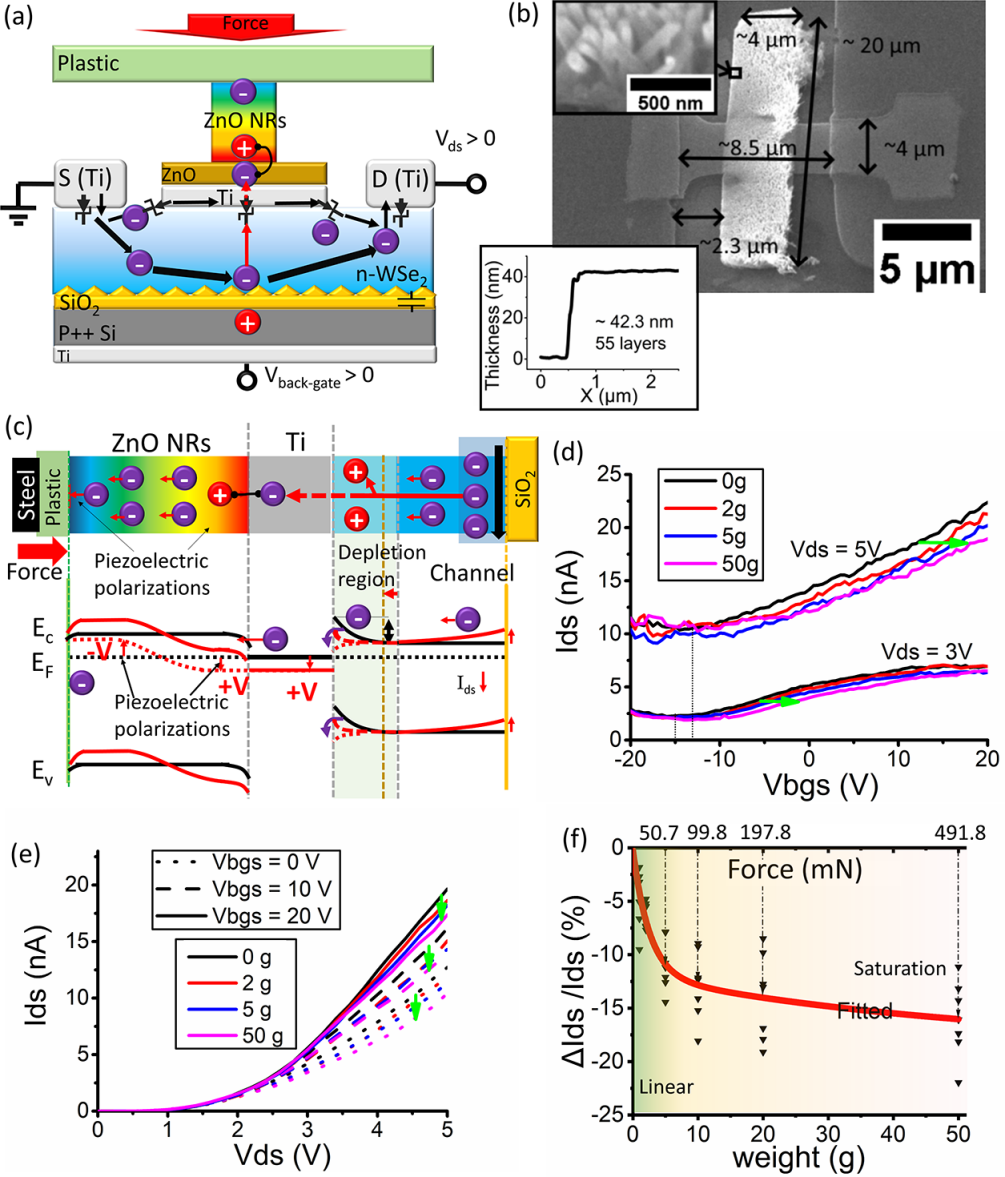
(a) Device structure and working mechanism schematic of the ZnO-Ti-WSe_2_ piezo-gated transistor. (b) SEM images of exampled ZnO-Ti-WSe_2_ piezo-gated transistor (inset with AFM profile showing the thickness of WSe_2_ flake, and the 45° tilted high magnification SEM images of ZnO NRs). (c) Energy diagram showing the piezo-gated Schottky barrier mechanism (red line is the band diagram after applying strain on ZnO NRs). (d) Transfer characteristics and (e) output characteristics of the ZnO-WSe_2_ piezo-gated transistor at different weight loadings. (f) Drain–source current change ((Δ*I*_ds_/*I*_ds_)%) as a function of applied weights; the scatter points are from both transfer and output characteristics.

The other device has been designed and presented (Dev. 2, see Figure 5), which includes an intermediate Ti metal layer (10 nm thick) between n-type WSe_2_ and ZnO. There are a few purposes for fabricating and presenting another force-sensing transistor device. First, the pristine WSe_2_ shows an ambipolar behavior,^49^ and the mechanically exfoliated WSe_2_ with different thicknesses can have different carrier types.^50^ A device with an n-type doped channel could have a different force-sensing behavior under the force loading compared to Dev. 1. The study of the different doping types of the force-sensing transistor channel may help with the future sensing logic system, similar to the “CMOS” system. Second, using Ti as a metal contact with WSe_2_, the contact type could be both Schottky and ohmic,^51^ depending on the doping level of WSe_2_. During our experiments, the Ti contact mostly shows a Schottky contact with WSe_2_. One purpose of Dev. 2 is to study the piezo-gated behavior with Schottky barrier modulation (see Figure 5a,c) and fabricate a piezo-gated “MESFET” (metal–semiconductor field-effect transistor). Meanwhile, the Ti adhesion layer has been used frequently to improve the adhesion of the seed layer and control the ZnO NRs morphology.^52^ Therefore, it is worthwhile to investigate the device with a Ti layer between the ZnO and the WSe_2_ channel. Furthermore, as a force sensor, the critical sensing block is the ZnO NR array. The area of the ZnO NR array and the sizes of ZnO NRs can change the force distribution. On the basis of the study of Dev. 1, the overall system can work under the force applied by less than 5 g of load. To increase the maximum mechanical force that could be applied, a ZnO NR array with a larger area could be integrated on the devices.

For the Dev. 2, a multilayer (∼55 layers) WSe_2_ transistor with n-type doped behavior has been selected before the growth the ZnO NRs. Then 10 nm Ti and 50 nm ZnO has been deposited as the seed layer. After hydrothermal growth, ZnO NRs of Dev. 2 (see SEM image in Figure 5b) have been observed to have a relatively small size (∼50 nm diameter, ∼700 nm length) and a high density with the same growth parameters as Dev. 1. The reason could be the smaller grains of the seed layer due to the existence of the Ti layer,^52^ together with the interactions among the less adjacent crystals, the weaker Gibbs–Thomson effect, and the more competitive growth^53^ due to a larger growth area (4 μm × 20 μm) than that of Dev. 1. With a smaller size of ZnO NRs and a larger growth area, the mechanical pressure on ZnO NRs could be smaller than that on Dev. 1. Therefore, larger weights can be loaded on Dev. 2 without breaking the ZnO NRs (up to 50 g or 0.5 N during the experiment).

For the electrical properties of the WSe_2_ channel in Dev. 2, the transfer characteristics of the overall Dev. 2 can be seen in Figure 5d. An n-type doped behavior has been observed when the back gate voltage is between −20 to 20 V. It is noticed that a large off-current and a low on–off ratio have been observed, and the *I*_ds_ has been found to have an almost linear relationship when *V*_bgs_ is between 0 to 10 V. The reason for the large off-current could be that the Ti metal layer on the top-side short circuits part of the WSe_2_ channel. From the output characteristics (Figure 5e), an apparent Schottky contact behavior has been observed where *I*_ds_ starts to increase until *V*_ds_ is larger than 2 V. Therefore, it can be confirmed also that Ti forms a Schottky contact with n-type doped WSe_2_ in Dev. 2.

For the force-sensing performance of Dev. 2, the strain applied by calibration weights has been observed to induce a positive shift of back-gated transfer characteristics to the n-side by 3–5 V (Figure 5d) and a decreasing of *I*_ds_ by up to ∼15% (Figure 5e). Figure 5f shows the percentage of *I*_ds_ change ((Δ*I*_ds_/*I*_ds_)%) under the different loading weights. The measurements clearly shows two phases: from 0 to 5 g, the *I*_ds_ changes linearly with the load increasing, which agrees with the linear relationship between the piezoelectric voltage and the applied strain; when the loaded weight is over 10 g, the *I*_ds_ change becomes saturated.

Although the main direction of the *I*_ds_ change in Dev. 2 (negative) is different from that in Dev. 1 (positive), the roles of piezo-gated ZnO NRs are similar. For both devices, the pressure induces a positive piezoelectric voltage on the bottom side of ZnO (top side of WSe_2_ or Ti) and then results in a positive shift on back-gate transfer characteristics. However, a significant difference is the doping type of the WSe_2_, which is believed to be the reason for the different direction of the *I*_ds_ change. The WSe_2_ of Dev. 2 is pristine n-typed doped, while the WSe_2_ in Dev 1 is observed to be p-typed doped possibly because of XeF_2_ overetching. In addition, the heterojunction control mechanism of Dev. 2 is believed to be based on the Schottky barrier modulation of Ti-WSe_2_, which is different from the reversed biased p–n junction control of Dev. 1. As shown in Figure 5a,c, there will be a depletion region on the WSe_2_ side of the Ti-WSe_2_ Schottky junction due to the different work functions. The electron could not flow freely from WSe_2_ to Ti without a positive voltage. When pressure is applied on ZnO NRs, the positive piezoelectric potential on the bottom side of ZnO could induce the electron flowing from Ti to ZnO (ohmic-contacted), which increases the electric potential of the Ti layer. Simultaneously, the increasing potential on the Ti layer could lower the Schottky barrier between Ti and WSe_2_, leading to the electron injection from WSe_2_ to Ti. The majority of the carriers (electrons) flow away from the conducting channel of the FET, resulting in the decreasing of *I*_ds_.

### 2.3 Discussion of Mixed-Dimensional Transistors as Force Sensors

Table 1 lists the different cases of field-effect transistors that have been gated by vertical ZnO NRs from both our experiments and others’ research.^14,22,24,46^ It is worth noting that, in addition to the presented devices, we have also fabricated one device which has n-type ZnO NRs directly on n-type WSe_2_ (Supporting Information Section S4).

**Table 1:** ZnO NR Piezo-gated Field-Effect Transistor

device	bottom of ZnO NRs	ZnO NR length, area	piezo-gated interface	top of ZnO NRs	detection range	sensitivity
ref (46)	MOS capacitor	∼8 μm, 1 mm ×1 mm	ZnO-Ag/Ti (Ohmic)	Kapton tape, metal probe	1–32 N, 0.1–3.2 MPa	+80 mV/8 N
ref (22)	N-MOSFET (p-Si)	∼800 nm, large area	ZnO-Al-SiO_2_-Si	Ceramic probe	Pressure not measured	+10% *I*_ds_ change
ref (24)	graphene	∼2 μm, large area	ZnO-graphene (Schottky)	Polyurethane (PU), metal probe	40–160 kPa	Dirac point shifts: 1.62 mV/kPa
ref (14)	2D MoS_2_ (N-type)	∼1 μm, 5 μm × 30 μm	ZnO-MoS_2_ (N–N)	Epoxy, metal probe	0.75–6.25 MPa	–25% *I*_ds_ change/6.25 MPa
our work (main content)	multilayer WSe_2_ (P-type)	∼6 μm, 2 μm × 4 μm	ZnO-WSe_2_ (N–P)	PP plastic plate, calibration weights	1–5 g, 10–50 mN, 0.1–0.5 kPa^a^	+30% *I*_ds_ change/5 g
	multilayer WSe_2_ (N-type)	∼700 nm, 4 μm × 20 μm	ZnO-Ti-WSe_2_ (Ohmic-Schottky)		1–50 g, 10–500 mN, 0.1–5 kPa^a^	–11% *I*_ds_ change/5 g
our work in Section S4	multilayer WSe_2_ (N-type)	∼2 μm, 9 μm × 16 μm	ZnO-WSe_2_ (N–N)	Parylene C, metal probe	1–10 N, 0.1 MPa–1 MPa^b^	–25% *I*_ds_ change/10 N

a The pressure of weights on Dev. 1 and Dev. 2 have been estimated by gravity force divided by the area of the PP square (1 cm^2^). However, the exact stress on the ZnO NRs could be in the scale of GPa, due to the small loading area of the tips of ZnO NRs.

b The pressure on the Parylene C coated device in Section S4 has been applied by a metal probe (2 mm × 5 mm).

As a result of the different device designs, encapsulations, and mechanical coupling, the output of the force-sensing transistor can be different. The force/pressure/strain sensing sensitivity of the integrated transistor could depend on the transfer characteristics of the semiconducting channel and the piezoelectric characteristics of the ZnO NRs. Generally, mechanical pressure on ZnO NRs leads to a positive potential on the top side of the interface between ZnO NRs and WSe_2_, which is equivalent to an additional negative voltage on the back gate. The direction of the *I*_ds_ change could be dependent on the channel material doping type and the charge transfer between the ZnO NRs and the transistor channel. In our case with pressure on ZnO NRs, an increasing *I*_ds_ has been observed in the channel p-type doped WSe_2_, while a decreasing *I*_ds_ has been observed in the n-type doped WSe_2_ channel.

The piezoelectric output could relate to the ZnO NRs themselves and the mechanical coupling. On the one hand, the ZnO NRs’ geometries and growth areas could possibly have an impact on the piezoelectric output.^46,54^ As shown in Table 1, apart from the different directions of the current change, the force sensing range and sensitivity may depend on the ZnO NRs’ areas. In our case, Dev. 2 is designed with a larger area of piezoelectric NRs compared to Dev. 1 and has been observed to possess a larger force sensing range but a lower sensitivity under the same force. On the other hand, the encapsulation layer (such as epoxy, PP plastic, Parylene C) could possibly have an influence by sharing strain and capacitance coupling,^46^ leading to the different degrees of output signals. For the encapsulation, as shown in Table 1, the device in Supporting Information Section S4 has a larger sensing range (1 to 10 N) by the Parylene C coating than the device with the PP plastic plate (Dev. 1 and Dev. 2).

In addition to the piezoelectric effect of ZnO NRs, the origin of charge redistribution in ZnO NRs could be possibly due to the contacting/friction interface charges between ZnO NRs and PP plastic^55^ or the electrical coupling between ZnO NRs and calibration weights (steel). Furthermore, 2D transition metal dichalcogenides (including MoS_2_ and WSe_2_) have been reported to possess a piezoelectric effect;^56−59^ however, the piezoelectric response of vertical ZnO NRs with a high aspect ratio is believed to be dominant for the output of the mixed-dimensional device.^14^ Moreover, the overall hybrid transistor can be sensitive to other environmental factors, such as gas and light (Supporting Information S3 and refs (23, 25, 26, and 34)), due to more complex mechanisms.

This work demonstrates the possibility of the 1D–2D transistors as a force sensing unit with the modification of carrier distribution in the channel by the piezoelectric effect of ZnO NRs. The force sensing performance of the 1D–2D transistors is relatively limited compared to mature force sensors, mainly due to the unstable mechanical/charge coupling between ZnO NRs and encapsulation layer, together with the inconsistent electrical performance of WSe_2_ after the hydrothermal growth at 90 °C. In addition, the top sides of the ZnO NRs are floated and in contact with the encapsulation layer directly, which could lead to unstable residue charges on the top gate of the transistor. The shifting between the ZnO NRs and the encapsulation layer under mechanical loadings could degrade the consistency of the force sensing, also resulting in relatively large hysteresis and noise. A grounded metal layer and a larger encapsulation layer could be desirable to improve the overall mechanical/charge coupling. In addition, a large-area deposition method of the 2D WSe_2_ with high quality and uniformity is still required to achieve the high-density integration and the force-mapping system.

Overall, with a more controlled process and systematic parametric study in the future, the hybrid transistor could be designed as a multisensing unit in future smart sensor networks or medical instruments.

## 3 Conclusions

In conclusion, we have proposed the concept and demonstrated the possibility of the 1D–2D transistor as a force-sensing unit by integrating 1D ZnO piezoelectric gate control on the multilayer WSe_2_ channel. Two example devices have been fabricated and discussed. The electrical properties of WSe_2_ FET have been found to play a significant role in the direction of *I*_ds_ change and the sensitivity of the overall force-sensing transistor. The pressure from a calibration weight of 5 g has been observed to result in ∼30% *I*_ds_ positive change for ZnO NRs on the p-type doped WSe_2_ device, and ∼10% *I*_ds_ negative change for the device with an n-type doped WSe_2_. The percentage change of *I*_ds_ ((Δ*I*_ds_/*I*_ds_)%) shows a dependence on the applied back-gate voltage, which provides the possibility of the integrated transistor as an active force sensor. The overall results show the possibility of the 1D–2D transistor as a nanoscale multisensing unit with tunable *I*_ds_ changing directions and sensitivities. With a more controlled process and systematic parametric study in the future, the hybrid transistor could be designed as a multisensing unit in smart sensor networks or medical instruments.

## 4 Materials and Methods

### 4.1 Fabrication of the WSe_2_ Transistor

Multilayer WSe_2_ has been exfoliated and transferred onto Si substrates with 300 nm SiO_2_. The WSe_2_ flake has been patterned with photolithography and XeF_2_ vapor etching (25 sccm vapor XeF_2_ with 100 sccm N_2_ at 1 Torr for 3–5 min). The RIE system with CHF_3_ has been used to label the location of the flakes by etching 25 nm thick of SiO_2_. After stripping the photoresist and cleaning the substrate by solvent NMP 1165 remover, another step of photolithography has been performed to define the area of the electrode. Electron beam evaporation has been used to deposit 100 nm thick Ti as the electrode together with the lift-off technique.

### 4.2 Integration of ZnO NRs on the WSe_2_ Transistor

First, photolithography has been performed to define the area for the ZnO seed layer and ZnO NRs. After exposing the area, electron beam evaporation has been performed to deposit 50 nm ZnO (Dev. 1) or 10 nm Ti/50 nm ZnO (Dev. 2) as the seed layer. It is worth noting that the ZnO seed layer has been found to be absent on photoresists without a Ti intermediate seed layer. Before the stripping of photoresist, ZnO NRs have been grown hydrothermally on the seed layer. The equimolar (1:1) zinc nitrate and HMTA have been mixed in the DI water (concentration: 40 mM) as the hydrothermal chemical solution. ZnO NRs have been grown at 90 °C for 3 h in the chemical solution. After the growth, the photoresist has been stripped, and the seed layer and NRs have been lifted off by NMP 1165 remover.

### 4.3 Characterization

The thickness of WSe_2_ has been measured by AFM, and the overall structure has been characterized by SEM. The electrical properties of the transistors have been measured before and after the integration of ZnO NRs. Before the force-sensing characterization, a PP plastic plate was put on the device for encapsulation. The steel calibration weights (1–5 g for Dev. 1 and 1–50 g for Dev. 2) have been used to apply the mechanical force on the device. All of the electrical characterizations have been performed by a Keithley 4200 semiconductor analyzer.

## Supporting Information Available

Section S1 describing a supplementary method for device fabrication, Section S2 providing details on the force-sensing characterization of Dev. 1, Section S3 showing the light intensity sensing measurement of Dev. 1, and Section S4 showing the supplementary device (ZnO–n-type doped WSe_2_ with Parylene C for encapsulation) (PDF)

## Abbreviations

FET: field-effect transistor
ZnO: zinc oxide
WSe_2_: tungsten diselenide
