# Supplementary material for: Mixed Dimensional ZnO/WSe2 Piezo-gated Transistor with Active Millinewton Force Sensing
Source: ACS Appl Mater Interfaces. 2022 Oct 19;14(43):49026–34. doi: 10.1021/acsami.2c15730 (PMC9634694; doi:10.1021/acsami.2c15730)
Supplement: Supplementary file 1 — am2c15730_si_001.pdf [file am2c15730_si_001.pdf]

Supporting information

Mix-dimensional ZnO/WSe_2 piezo-gated transistor with active millinewton force-sensing

Author Information

Yulin Geng[1 (*)], Jing Xu[1], Muhammad Ammar Bin Che Mahzan[1], Peter Lomax[1], Muhammad Mubasher Saleem[2], Enrico Mastropaolo[1(†)], and Rebecca Cheung[1]

Author affiliations

[1] Institute for Integrated Micro and Nano Systems, School of Engineering, University of Edinburgh, Scottish Microelectronics Centre, Edinburgh, EH9 3FF, United Kingdom

[2] Department of Mechatronics Engineering, National University of Sciences and Technology (NUST), Islamabad, 44000, Pakistan

(†) Deceased 15 July 2019

(*) Corresponding author email address: yulin.geng@ed.ac.uk

Supplementary Information

S1. Supplementary method: Device fabrication

S2. Details on the force-sensing characterization of Dev.1

S3. Light intensity sensing measurement of Dev.1

S4. Supplementary device (ZnO - n-type doped WSe_2 with Parylene C as encapsulation)

S1. Supplementary method: Device fabrication

The fabrication of the 1D-2D transistor includes the fabrication of 2D WSe_2 FET and the integration of ZnO NRs on top of WSe_2, as shown in **Figure S1**. Mechanical exfoliated WSe_2 flakes have been selected as the channel of the transistor. 2D WSe_2 FET has been fabricated by photolithography; the details of the photolithography steps on the 2D WSe_2 channel patterning and electrode patterning have been presented.

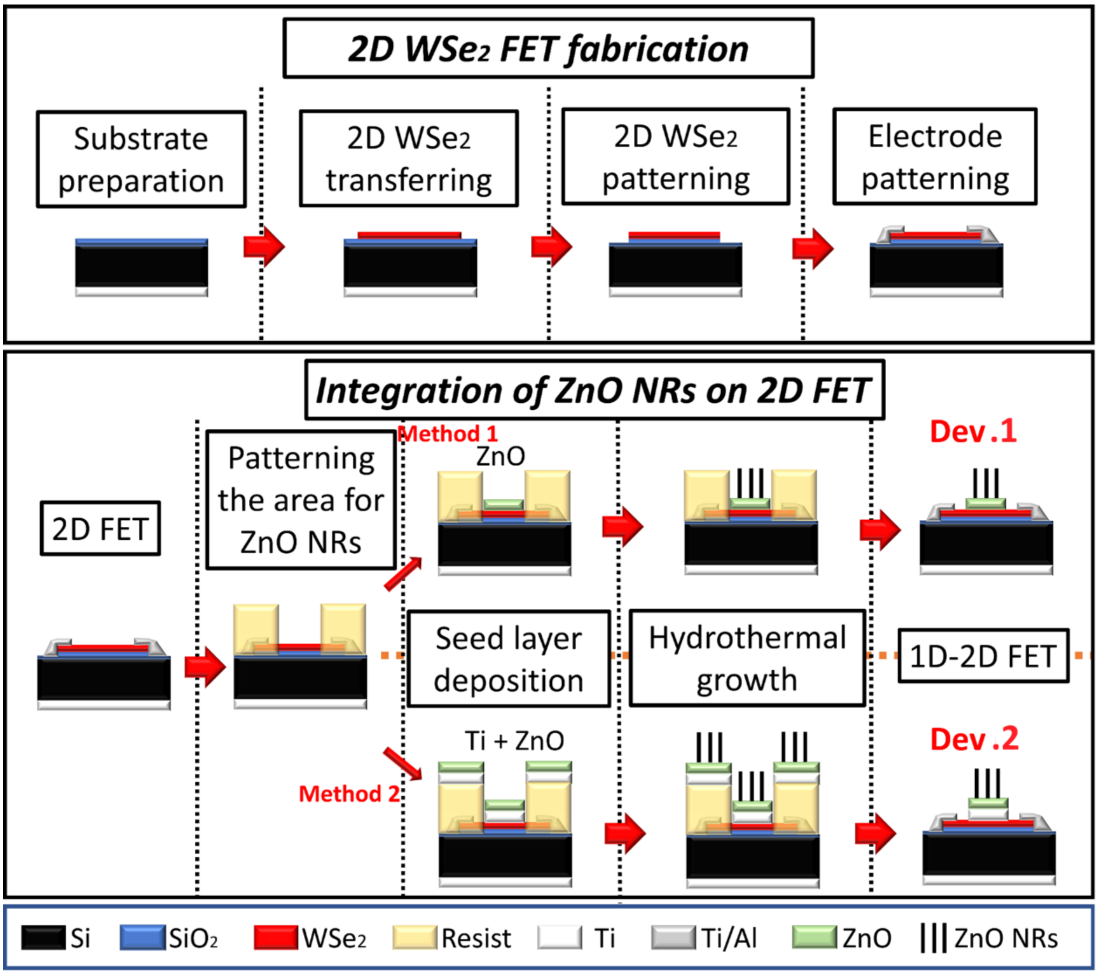

Figure S1 Schematic showing the fabrication process of 1D-2D transistors.

The optical images of the 2D WSe_2 FETs on both devices have been presented in **Figure S2**. The first device (Dev.1) has an over-etched WSe_2 channel (see **Figure S2 (b)**), which could have a p-typed doped behaviour due to the edge defects induced by the XeF_2 vapour[1]. As for Dev.2, pristine WSe_2 has been selected as a channel of FET, which could be n-doped FET. The NRs lift-off method has been used for the patterning of ZnO NRs on WSe_2 FET. Therefore, for Dev.2, there is a Ti metal layer between the ZnO seed layer and the WSe_2 channel.

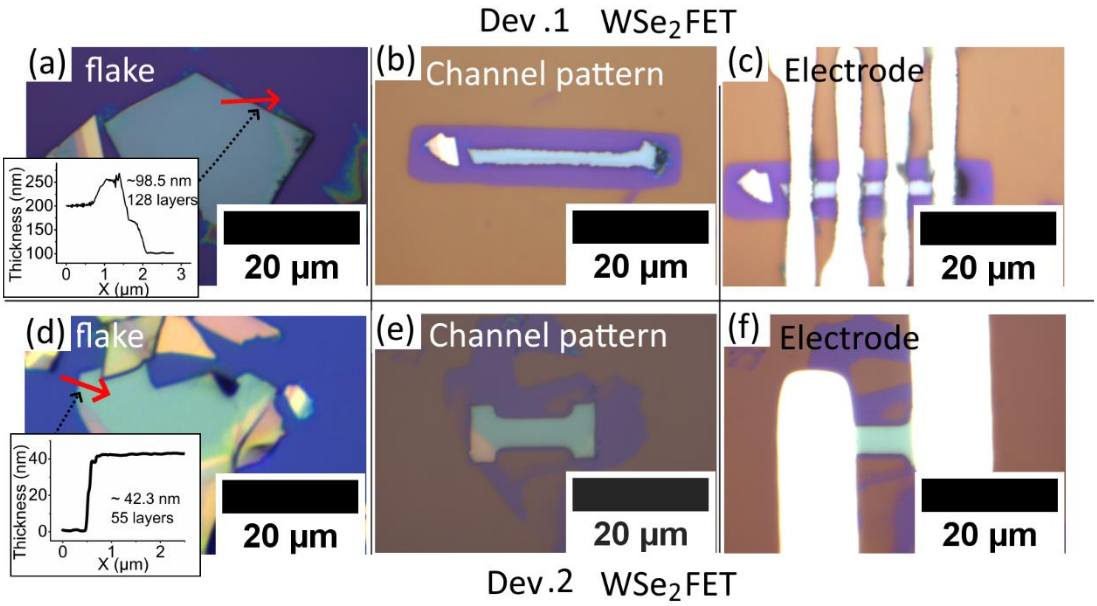

Figure S2 Optical images of 2D WSe_2 FETs during fabrication, Dev.1 (a-c) and Dev.2(d-f). (a,d) selected WSe_2 flakes and inserted with AFM line scan profiles; (b,e) The WSe_2 channel patterned with XeF_2 vapour etching and RIE labelling; (c,f) The electrode of WSe_2 FETs.

The P++ Si has been used as the substrate and the back-gate, with 300 nm thick SiO_2 as the back-gate dielectric and 100 nm thick Ti as the back-gate contact. WSe_2 flakes have been exfoliated mechanically and transferred to the SiO_2 by the scotch tape. A relatively large flake has been selected for the channel material, as shown in **Figure S2 (a) and (d)**. The thickness of the WSe_2 flake has been measured by AFM, which is ~98.5 nm for Dev.1 and ~42.3 nm for Dev.2. Photolithography has been performed to define the channel geometry, followed by 25 sccm vapour XeF_2 with 100 sccm N_2 at 1 Torr for 3-5 mins. The thicker flake takes a longer time to be

etched away. However, as the etching time increases, the XeF_2 vapour can go underneath the photoresist and etching the side of WSe_2 protected with photoresist, resulting in the over-etching of the WSe_2 flake on the edge (Dev.1, see **Figure S2 (b)**). In literature, the edge etching by XeF_2 or RIE could induce defects and then result in the p-type doping of the WSe_2 flakes[1]. After the XeF_2 etching, JLS RIE etching (CHF_3/Ar plasma) has been performed to etch ~ 25 nm SiO_2 as the label marker. The resulting WSe_2 channel widths have been measured to be 2 µm for Dev.1 and 4 µm for Dev.2, as shown in **Figure S2 (b) and (d)**.

After the WSe_2 flakes patterning, a second photolithography process has been performed to fabricate the electrode. Negative resist AZ2035 has been patterned, and the area for the electrode has been exposed. E-beam evaporation has been used to deposit 10 nm Ti and 300 nm Al as contact metal. Lastly, the lifting-off of the metal has been achieved by remover NMP1165 at 70 °C for several hours. As shown in **Figure S2 (c) and (f)**, the resulting WSe_2 FET of Dev.1 has been achieved to has a channel length of 3×4 µm (4 electrodes) and a channel width of 2 µm. For Dev.2, the channel length has been achieved to be 8.5 µm (only two electrodes), and the channel width has been measured to be 4 µm. Before integrating ZnO NRs on 2D FETs, Keithley 4200 semiconductor analyzer has been used to characterize the electrical performance of 2D WSe_2 FETs.

As for the integration of ZnO NRs on the 2D WSe_2 channel, the e-beam evaporated ZnO thin-films (without Ti adhesion layer for Dev.1 and with Ti Dev.2) have been used as the seed layer of ZnO NRs. A simplified method[2] has been performed to integrate ZnO NRs on the WSe_2 FET of Dev.1, where the ZnO NRs and ZnO seed layer are contacted directly with the WSe_2 channel without a Ti adhesion layer. The omitted deposition of a Ti adhesion layer has been found to result in the absence of the ZnO seed layer on photoresist[2]**.** The positive photoresist SPR 350 has been used to define the area for seed layer deposition and ZnO NRs growth. The thickness of SPR 350 has been measured to be ~1.5 µm. The designed geometry for Dev.1 is 1.5 µm ×10 µm, while 4 µm × 20 µm for Dev.2. Then e-beam evaporation has been used to deposit 50 nm ZnO for Dev.1 and 10 nm Ti/50 nm ZnO for Dev.2.

After the seed layer deposition, ZnO NRs has been grown hydrothermally at 90 °C for 3 hours, with a precursors concentration at 20 mM. Then, acetone has been

used to strip the photoresist. It is worth noting that, for Dev.2, it takes a relatively long time to lift off the ZnO NRs on the photoresist. The resulting ZnO NRs have been characterized by SEM and shown in **Figure S3**. A cluster of flower-like ZnO NRs array on Dev.1 has been observed, and ZnO NRs have been found to possess a relatively large diameter (~1.3 μm) and long length (~5.9 μm). However, for Dev.2, very small and dense ZnO NRs has been observed, with a diameter of ~60 nm and a length of ~800 nm.

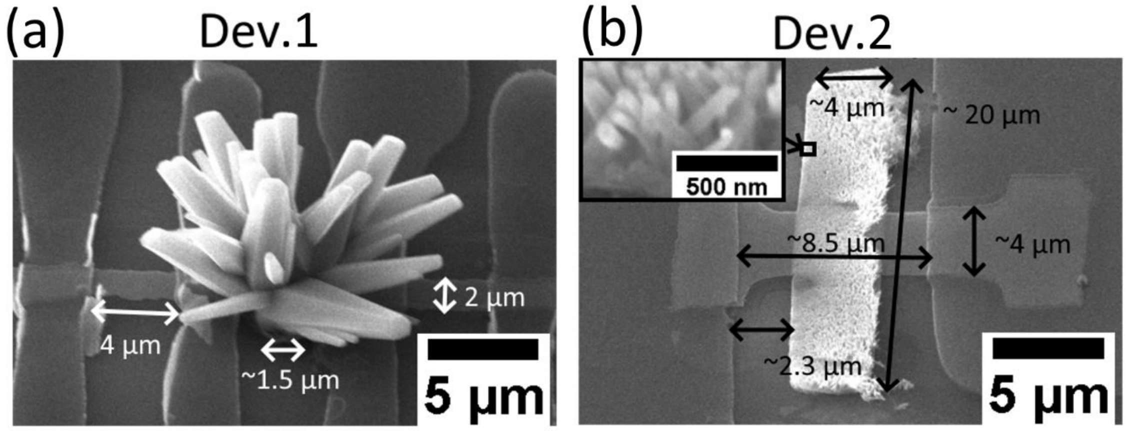

Figure S3. 45°tilted SEM images of 1D-2D transistor: (a) Dev.1 and (b) Dev.2, inset with high magnification SEM images showing ZnO NRs.

.

S2. Details on the force-sensing characterization of Dev.1

Figure S4 (a) shows the force-sensing testing structure of Dev.1. The Keithley 4200 semiconductor analyzer has performed the electrical characterization. The source and drain electrodes of the transistor are directly connected with the probe, while the back gate contact has been in contact with the metal stage with the vacuum, and then another probe has been used to contact with the metal stage.

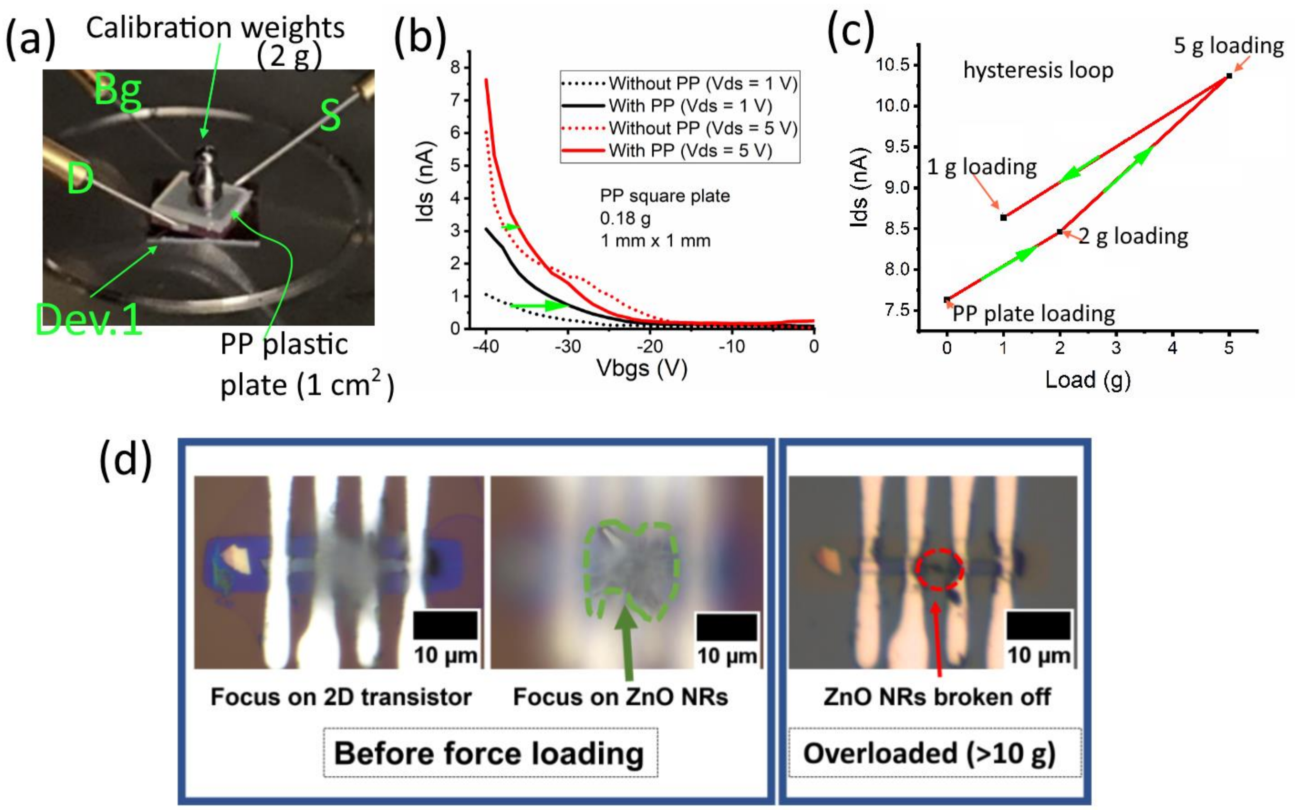

Figure S4 (a) Force testing structure; (b) The effect of 0.18 g PP plastic plate loading on the transistor current; (c) Loading sequence and hysteresis observation; (d) Optical images showing ZnO NRs before and after overloading.

The mechanical force has been applied manually by loading the steel calibration weights on a PP plastic plate (0.18 g). The PP plastic plate has been loaded

before the calibration weights. The transfer characteristics before and after the loading of the PP plastic plate have been presented in **Figure S4 (b).** The loading of the PP plate has been observed to increase the drain-source current from 6 nA to 7.5 nA (V_{bgs} = -40 V, V_{ds} = 5 V). In addition, if a lower V_{ds} has been applied (1 V), the I_{ds} change has been observed to be more significant (from 1 nA to 3 nA). The large force sensing response of 0.18 g load may indicate that ZnO NRs may have a linear region between 0 g to 1 g, and the loads over 1 g have already been in the saturation region. In addition, the contact charge between ZnO NRs and PP plate could be the other origin of the large I_{ds} change.

After loading the PP plate, the different calibration weights were loaded. In the main content, the I_{ds} change under different loads has been calculated by the Ids under weights minus the I_{ds} when PP plate are already loaded to eliminate the effect of the loading of the PP plate. The manually loading sequence has been set as 2 g, 5 g and 1 g. The transfer curve has been recorded for each weight loading, and the time interval between different loading is about 5 min. The I_{ds} curves (V_{bgs}= - 40V and V_{ds} = 5 V) as a function of different loads have been plotted in **Figure S4 (c)**. The force loading between 1 g loading after 5 g loading is at the same level as the first time 2 g loading, which could indicate the force-sensing hysteresis and suggest that a more stable mechanical structure design and encapsulation may be required in the future.

To investigate the maximum loading limit of Dev.1, the higher weight (over 10 g) has been attempted. However, the device has been observed to be broken; the ZnO-WSe_2 has been seen to be peeled from the substrate (**see Figure S4 (d)**). The reason could be the ZnO NRs' overloading or the shear force between ZnO NRs and PP plastic plate. The maximum loading limit could be increased by the larger ZnO NRs growth area and a smaller ZnO NRs; however, the overall sensitivity could be decreased, as described in the main content (Dev.2).

S3. Light intensity sensing measurement of Dev.1.

During the electrical and force-sensing characterization of the 1D-2D transistor, the different intensity of light has been found to influence the I_{ds} of the transistor. For Dev.1, the light intensity sensing capability has been characterized by the probe station with an adjustable intensity lamp. The wavelength spectrum of the light from the lamp has been measured by a spectrometer, as shown in **Figure S5(a)**. The light applied by the lamp includes two groups of main peaks at the wavelength at 360-510 nm (blue) and 600- 700 nm ranges (orange).

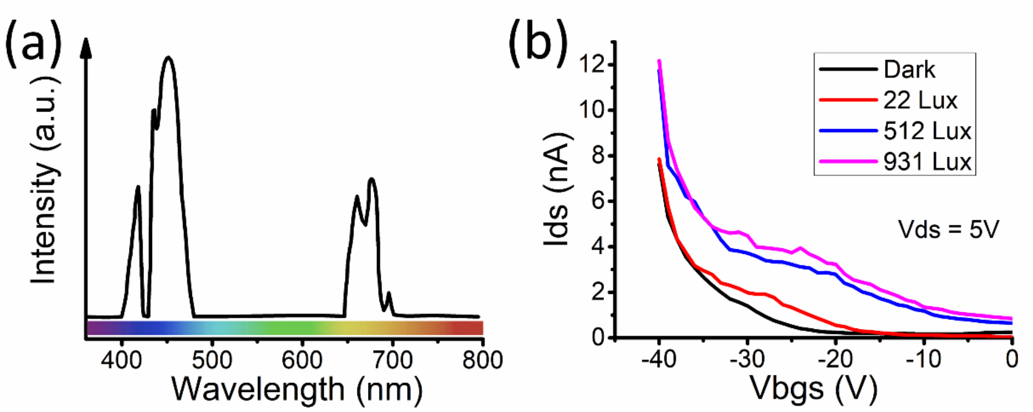

Figure S5. (a) Wavelength spectrum of the light from the lamp;(b) transfer characteristic of 1D-2D transistor under different light intensity.

The light intensity has been measured by a lux meter, and the transfer characteristic of the Dev.1 has been presented in **Figure S5 (b)**. The sensitivity of the devices to light is relatively high, and the maximum I_{ds} change has been found to be ~ 6 nA under the light with the intensity of 931 lux (when the I_{ds} in the dark is ~6 nA, V_{ds} = 5V, V_{bgs} =-40V). It is believed that the light-sensing ability of the transistor is due to the photoelectric effect of the WSe_2 channel[3]. The overall transistor could act as a phototransistor for blue light or orange light. It is worth noting that, after encapsulated with Kapton tape (yellow colour, polyimide), the light-sensing ability of the transistor has been found to be shielded.

S4. Supplementary device (ZnO – n-type doped WSe_2 with Parylene C as encapsulation)

In addition to the Dev.1 and Dev.2 presented in the main content, another device with ZnO NRs directly grown on n-type doped WSe_2 (no Ti intermediate layer) has been presented in **Figure.S6**.

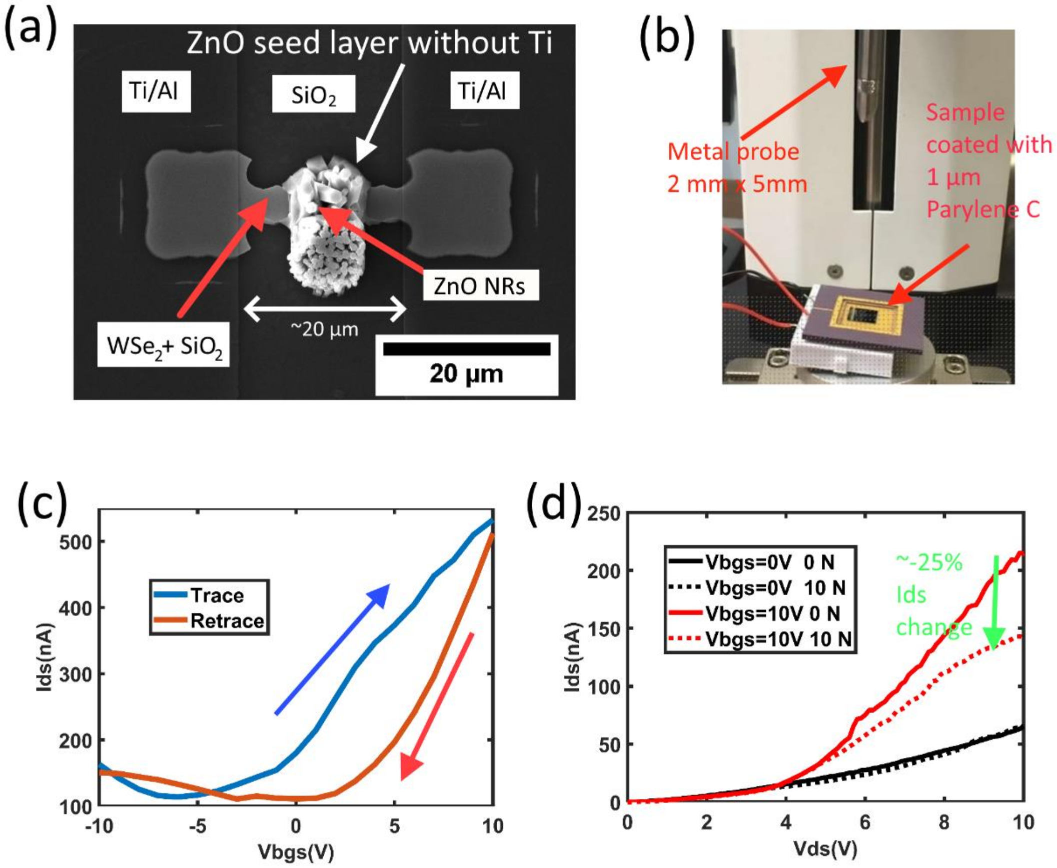

Figure S6. (a) SEM images of ZnO-WSe_2 (n-type) transistor, WSe_2 channel length = 20 μm, width = 5 μm, ZnO area = 9 μm×16 μm, NWs diameter ~500 nm, length~2 μm; (b) Force sensing testing set-up by force gauge, metal probe size: 2 mm×5 mm, sample wire-bonded and encapsulated with 1 μm Parylene C; (c) transfer characteristic of the transistor (V_{ds} = 10 V); (d) Output characteristic of under mechanical force loading;

The fabrication of the supplementary device is similar to Dev.1, which has been presented in **Supplementary S1**. The resulting structure of the supplementary device can be seen in **Figure S6 (a)**. Vertically aligned ZnO NRs have been observed to grow on the etched WSe_2 channel, where ZnO thin-film (no Ti adhesion layer) has been used as the seed layer. After the fabrication, the device has been coated with 1 µm Parylene C as the encapsulation layer on top of ZnO NRs. A force gauge has been used to apply mechanical force on the device, and the overall diagram can be seen in **Figure S6 (b)**. Due to the force sharing by Parylene C in the surrounding of the ZnO NRs, the actual force that can be applied without breaking the device has been found to be larger than 10 N.

The transfer and output characteristics of the supplementary device have been presented in **Figure S6 (c) and (d)**. As can be seen, the overall transistor shows an n-type doped behaviour, with a threshold back-gate voltage of ~-5 V to 5 V. For the force sensing measurement, it can be seen in Figure.S5 (d) that the 10 N force applied on ZnO NRs could decrease the channel current. If the V_{bgs} is fixed at 10 V (linear region of the transfer curve), the I_{ds} has been observed to decrease up to ~25% by the 10 N mechanical force.

The force-related I_{ds} direction change of the supplementary device agrees with Dev.1 and Dev.2 in the main content. The changing direction of I_{ds} could be dependent on the doping type of the WSe_2 channel: for n-type doped WSe_2, the applied force on ZnO NRs piezo-gate could decrease the I_{ds}; while p-type doped WSe_2 could have an increase I_{ds} when a force is applied on ZnO NRs. Moreover, the supplementary device has different encapsulation and force-sensing setups. By using a thin encapsulation layer (Parylene C) instead of PP(polypropylene) plastic plate, the sensing force can possibly increase from a few mN to a few tens of N.

References in supplementary information

1. Zhang, R., Drysdale, D., Koutsos, V. & Cheung, R. Controlled Layer Thinning and p-Type Doping of WSe2 by Vapor XeF2. *Adv. Funct. Mater.* **27**, 1702455 (2017).

2. Geng, Y. *et al.* Simplified patterning process for the selective 1D ZnO nanorods growth. *J. Vac. Sci. Technol. B* **38**, 012204 (2020).

3. Wang, T. *et al.* High-Performance WSe2 Phototransistors with 2D/2D Ohmic Contacts. *Nano Lett.* **18**, 2766–2771 (2018).
